# Efficacy of clomifene citrate for the treatment of patients with polycystic ovary syndrome: A protocol of systematic review

**DOI:** 10.1097/MD.0000000000020590

**Published:** 2020-06-19

**Authors:** Qin-wei Han, Jin-ping Wu, Ying Pang, Li-xia Wu, Li-na Yang

**Affiliations:** a Department of Obstetrics and Gynecology, 521 Hospital of Norinco Group; b Department of Obstetrics and Gynecology, Institute for Hygiene of Ordnance Industry, Xi’an, Shaanxi, China.

**Keywords:** clomifene citrate, efficacy, Polycystic ovary syndrome, safety

## Abstract

**Background:**

This study aims to assess the efficacy and safety of clomifene citrate (CC) for the treatment of patients with polycystic ovary syndrome (PCOS).

**Methods:**

In this study, we will comprehensively search MEDLINE, EMBASE, The Cochrane Library, Web of Science, CINAHL, ACMD, PsycINFO, and China National Knowledge Infrastructure for original articles published from their inceptions to the January 1, 2020 without language restrictions. All studies will undergo relevance and a design selecting process. Data from qualified studies will be collected by 2 independent authors. Additionally, we will conduct a risk of bias evaluation using a Cochrane risk of bias tool. We will undertake statistical analysis utilizing RevMan 5.3 software.

**Results:**

This study will summarize the up-to-date evidence to investigate the efficacy and safety of CC for the treatment of patients with PCOS.

**Conclusion:**

The findings of this study will provide helpful evidence of CC for the treatment of patients with PCOS, as well as may help develop treatment guidelines.

**PROSPERO registration number:**

PROSPERO CRD42020162818.

## 1 Introduction

Polycystic ovary syndrome (PCOS) is one of the most prevalent endocrine disorders in reproductive-aged females,^[1–3]^ affecting 5% to 15% of those females.^[4–8]^ It is reported that its incidence in females with anovulatory infertility ranges from 70% to 80%.^[9]^ It is characterized by biochemical hyperandrogenemia, chronic anovulation, and polycystic ovaries.^[4,5,10–12]^ Despite a number of treatments have been developed to manage PCOS, the outcome results are still unsatisfactory.^[13–17]^ Thus, it is necessary to develop more medications for PCOS in high demand.

Previous studies showed that clomifene citrate (CC) has effects in inducing ovulation and benefit for pregnancy.^[18–26]^ However, there is no systematic review that specifically focuses on investigating the efficacy and safety of CC for the treatment of patients with PCOS. Thus, the target of this study is to systematically assess the efficacy and safety of CC for the treatment of PCOS.

## 2 Methods and analysis

### 2.1 Study registration

This study has registered on PROSPERO (CRD42020162818). It will be conducted based on the guidelines of Preferred Reporting Items for Systematic review and Meta-Analysis Protocols.^[27,28]^

### 2.2 Eligibility criteria

#### 2.2.1 Type of studies

This study only considers randomized controlled trials (RCTs) for inclusion, which assess the efficacy and safety of CC for the treatment of patients with PCOS regardless language and publication status.

#### 2.2.2 Type of participants

Only female adults (>18 years’ old) who were diagnosed as PCOS will be included in spite of country, race, and educational background.

#### 2.2.3 Type of interventions

The treatment schedule in the experimental group must be CC assigned to all qualified patients.

The intervention schedule in the control group could be any treatments. However, any single or combination therapy of CC will not be considered in this study.

#### 2.2.4 Type of outcomes

The primary outcomes consist of sex hormone levels. It includes luteinizing hormone (LH), follicle stimulating hormone (FSH), LH/FSH ratio, androstadiendione, dehydroepiandrosterone sulfate, testosterone, and estradiol.

The secondary outcomes are total response rate, ovulation rate, and pregnancy rate, oral glucose tolerance test, body mass index (calculated using weight [kg]kilogram/height [square meters]), and adverse events.

### 2.3 Search strategy

We will undertake a thorough and rigorous search of the present literature sources from the following electronic databases from their inceptions to the January 1, 2020: MEDLINE, EMBASE, The Cochrane Library, Web of Science, CINAHL, ACMD, PsycINFO, and China National Knowledge Infrastructure. We will not implement any limitations of language and publication status. We will create a detailed search strategy for MEDLINE and will be presented in Table 1. In addition, we will adapt similar search strategies with details for other electronic databases.

**Table 1:**
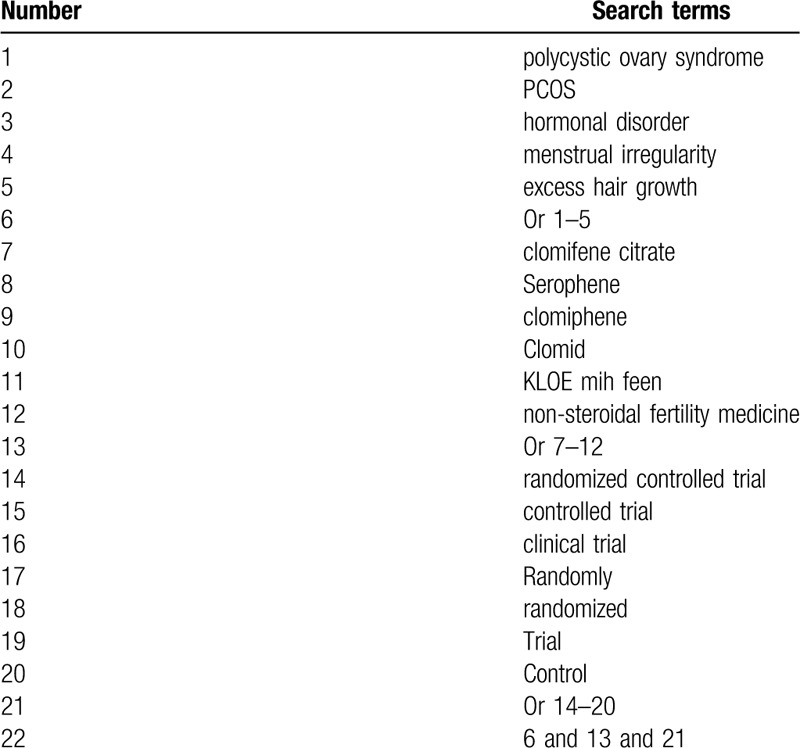
Search strategy of MEDLINE.

Further searches will be performed for conference proceedings, theses or dissertations, or reference lists of included studies or relevant reviews.

### 2.4 Data collection and management

#### 2.4.1 Study selection

Two authors will independently select studies against previously designed eligibility criteria. It includes 2 stages. First, all searched records will be checked by scanning titles and abstracts, and any unconnected studies will be removed at this stage. At the second stage, we will obtain full articles of all remaining studies based on the full inclusion criteria. We will document reasons for all removed studies at different stages. Any conflicts regarding the study selection between 2 authors will be resolved by a third author through discussion. The whole process of study selection with detailed information will be presented in the flowchart.

#### 2.4.2 Data extraction

All relevant data will be independently extracted by 2 authors based on the standardized database structured sheet that will be prepiloted with a sample containing at least three eligible studies. We will invite a third author for consultation or arbitration in case of any divergences between 2 authors. We will focus on the following items for data extraction:

1. Descriptive characteristics of the trial: country, first author, year of publication, trial setting, among others
2. Patient descriptive characteristics: race, age, diagnostic criteria, eligibility criteria, sample size, among others
3. Methods: trial design, detailed information of randomization, blind, among others
4. Interventions and comparators: intervention types, dosage, frequency, among others
5. Outcomes: primary and secondary outcomes, adverse events, among others
6. Any other relevant data: any associated information, including funding information, conflict of interests, among others.

If there is any information missing or unclear, we will contact the primary authors to request them. If we cannot get reply, we will perform analysis only based on the current available data.

### 2.5 Risk of bias assessment

Two authors will independently investigate the risk of bias for each qualified trial using Cochrane risk of bias tool. It covers 7 aspects and each one is specifically graded as low, unclear, or high risk of bias. Any conflicts of risk of bias assessment between 2 authors will be solved by a third author through discussion.

### 2.6 Statistical analysis

We will perform statistical analysis employing RevMan 5.3 Software.

#### 2.6.1 Data synthesis

In this study, we will occupy standardized mean difference and 95% confidence intervals (CIs) for continuous data, and risk ratio and 95% CIs for dichotomous data. We will examine the presence of statistical heterogeneity using *I*^2^ statistic. Its values will be interpreted as follows: *I*^2^ ≤ 50% implying low heterogeneity, while *I*^2^ > 50% suggesting obvious heterogeneity. If *I*^2^ ≤ 50%, we will exert a fixed-effects model, and meta-analysis will be performed if sufficient data are collected. However, if *I*^2^ > 50%, we will employ a random-effects model, and subgroup analysis will be operated to inspect the possible causes that may result in obvious heterogeneity. We will incorporate the quantitative data for each outcome of sex hormone levels (including such as LH, FSH, LH/FSH ratio, androstadiendione, dehydroepiandrosterone sulfate, testosterone, and estradiol), oral glucose tolerance test, and body mass index. We will integrate qualitative data for each outcome of total response rate, ovulation rate, and pregnancy rate, and incidence of adverse events.

#### 2.6.2 Subgroup analysis

We will preside over subgroup analysis in accordance with the different types of interventions and comparators, descriptive characteristics of the trial, characteristics of patient, interventions and comparators, and outcomes.

#### 2.6.3 Sensitivity analysis

In the case of sufficient data, sensitivity analysis will be handled to identify the stability of merged outcome results by deleting high risk of bias trials.

#### 2.6.4 Reporting bias

If we include at least 10 eligible trials, we will carry out Funnel plot and Egger regression test to explore whether there are any reporting biases.^[29,30]^

### 2.7 Quality of evidence

The quality of evidence for each outcome measurement will be evaluated by using Grading of Recommendations Assessment Development and Evaluation.^[31]^ It consists of five aspects and each one is specifically classified as high, moderate, low, or very low.

### 2.8 Ethics and dissemination

This study does not inquire ethical approval because no individual patient data from primary studies will be utilized. We plan to publish this study through a peer-reviewed journal or conference meeting.

### 2.9 Patient and public involvement

Patients and/or the public were not directly joined in the development of this study protocol.

## 3 Discussion

To date, an increasing number of clinical studies have reported the efficacy and safety of CC for the treatment of patients with PCOS; however, its efficacy and safety have not been fully investigated at literature level. To our best knowledge, this study will be the first one to summarize the most recent evidence to assess the efficacy and safety of CC for the treatment of patients with PCOS. It will perform a rigorous and comprehensive approach without language and publication time restrictions. The findings of this study may provide reference for both clinical practice and patients.

## Author contributions

**Conceptualization:** Qin-wei Han, Li-xia Wu, Li-na Yang.

**Data curation:** Li-xia Wu, Li-na Yang.

**Formal analysis:** Qin-wei Han, Jin-ping Wu, Li-xia Wu, Li-na Yang.

**Investigation:** Li-na Yang.

**Methodology:** Qin-wei Han, Jin-ping Wu, Ying Pang, Li-xia Wu.

**Project administration:** Li-na Yang.

**Resources:** Qin-wei Han, Jin-ping Wu, Ying Pang, Li-xia Wu.

**Software:** Qin-wei Han, Jin-ping Wu, Ying Pang.

**Supervision:** Li-na Yang.

**Validation:** Qin-wei Han, Jin-ping Wu, Ying Pang, Li-xia Wu, Li-na Yang.

**Visualization:** Qin-wei Han, Li-na Yang.

**Writing – original draft:** Qin-wei Han, Jin-ping Wu, Ying Pang, Li-xia Wu, Li-na Yang.

**Writing – review & editing:** Qin-wei Han, Jin-ping Wu, Ying Pang, Li-na Yang.
